# Invasive *Aspergillus* outbreak in inhalation injury: a case presentation and literature review

**DOI:** 10.1186/s12879-022-07366-7

**Published:** 2022-04-18

**Authors:** Shengli Liu, Zonghang Li, Jiansheng Zheng, Ning He

**Affiliations:** 1 Department of Burns and Plastic Surgery, The Affiliated Dongnan Hospital of Xiamen University, School of Medicine, Xiamen University, Zhangzhou, China; 2 School of Medicine, Xiamen University, Xiamen, China; 3 Intensive Care Unit, The Affiliated Dongnan Hospital of Xiamen University, School of Medicine, Xiamen University, Zhangzhou, China

**Keywords:** Inhalation injury, Pulmonary aspergillosis, Pathological diagnosis, Treatment, Prognosis

## Abstract

**Background:**

Invasive pulmonary aspergillosis often occurs in patients with poor immune function, who abuse steroids or broad-spectrum antibiotics, or who use intravenous drugs. Among the *Aspergillus* genus of pulmonary infection, *Aspergillus fumigatus* is the most important pathogen, followed by *Aspergillus flavus*, *Aspergillus niger*, and *Aspergillus terreus.* Inhalation injury complicated by *Aspergillus* infection has atypical clinical manifestations. Diagnosis is difficult, and it is easy to make mistakes in treatment. Moreover, there are few cases of burn inhalation injury complicated with pulmonary *Aspergillus*.

**Case presentation:**

We report a case of severe burns combined with severe inhalation injury, early pulmonary aspergillosis, and severe respiratory failure due to treatment discontinuation. Through analyzing the processes of diagnosis and treatment in the present case and performing a literature review, we explore feasible diagnosis and treatment plans.

**Conclusions:**

Early application of a variety of diagnostic measures can be used to identify *Aspergillus* infection, and targeted anti-infection treatment is likely to reverse a severe adverse prognosis.

## Background

Inhalation injury is one of the high-risk factors for invasive fungal infection after burns, with an incidence rate of < 1%. However, the mortality rate is > 50% [1] and can be as high as 79% in the intensive care unit [2]. Current research shows that fungal infection after inhalation injury is increasing each year; thus, it is attracting increasing attention. However, reported cases of *Aspergillus* infection after inhalation injury are extremely rare. This may be related to the unclear clinical manifestation of pulmonary aspergillosis [3]. Moreover, it is difficult to perform effective diagnostic examinations [4]. Thus, burn specialists’ knowledge of pulmonary aspergillosis is insufficient. In this study, we report a case of pulmonary aspergillosis in a patient with severe inhalation injury. Before pulmonary *Aspergillus* infection was determined, the patient presented with progressive hypoxemia. Tracheotomy, ventilatory support, and preventive antifungal therapy did not improve the patient’s respiratory condition. Finally, the patient discontinued treatment due to severe hypoxemia and died. The tortuous process of diagnosis and treatment may reflect the current treatment dilemma of inhalation injury complicated with pulmonary aspergillosis. As well as describing the case, we reviewed the current literature in an attempt to identify an effective solution to this problem.

## Case presentation

**Fig. 1:**
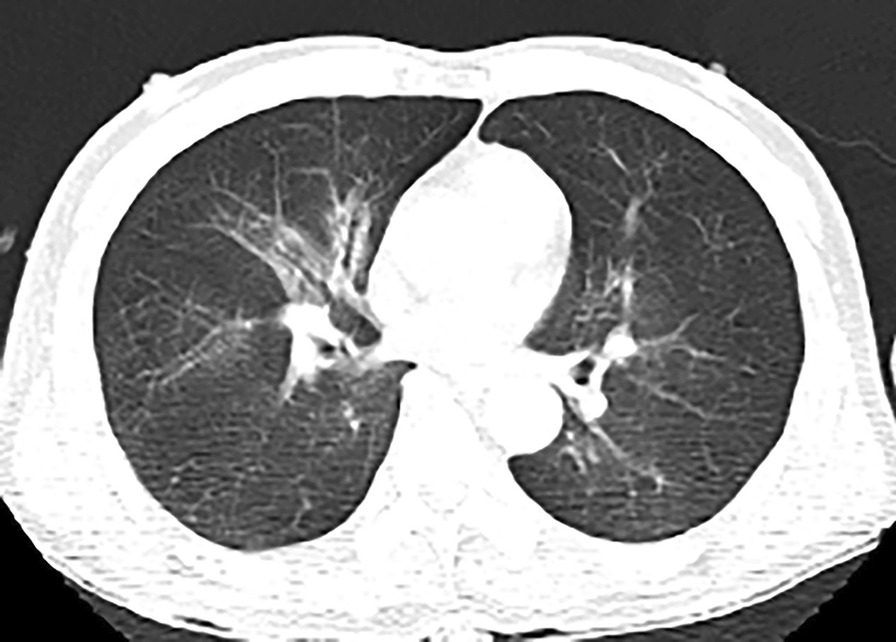
Slightly high-density, patchy, blurry shadows in bilateral bronchi, indicating inhalation injury

A 67-year-old male boiler worker (height, 170 cm; weight, 60 kg) was hospitalized on 31st January 2021, with full-body swelling and pain after thermal boiler ash burns with dyspnea for 4 h. A physical examination on admission revealed a clear mind, irritability, a regular heart rhythm, thick lung sounds, moist rales, and a soft abdomen. The patient had a 40-year smoking history and smoked 20 cigarettes per day. He frequently experienced cough and sputum. The patient had no history of other diseases. A burns specialist identified burns of the head and facial hair, burns of the nostril hair, throat swelling, III° area of approximately 70%, and a deep II° area of 18%. The patient had a long-term drinking habit and consumed 50 ml of alcohol per day. Emergency chest computed tomography (CT) findings were consistent with imaging findings of severe inhalation injury (Fig. 1). According to the history of burns obtained from hot furnace ash, skin burn manifestations, symptoms of dyspnea, and chest CT, a diagnosis of extremely severe burns of 88% of total body surface area (TBSA), severe inhalation injury, and burn shock was concluded, but the previous existence of pulmonary inflammation was still not clear.

Tracheotomy was performed immediately after admission. Continuous high-flow oxygen was administered to ensure an adequate oxygen supply. Moreover, a sufficient amount of lactate Ringer’s solution, 5% glucose, frozen plasma, and human blood albumin was combined with anti-shock treatment. During the first 2 days, the patient was administered piperacillin and tazobactam (4.5 g three times per day). On the third day, this regimen was adjusted to linezolid (0.6 g twice/day), meropenem (1 g thrice/day), and itraconazole (200 mg once/day). The gastric mucosa was protected by omeprazole (40 mg/day). Sulfadiazine silver powder was coated on the wound surface of the whole body, treated by exposure to red light, and placed on a suspension bed. Urine volume and arterial pressure were monitored during treatment to maintain hemodynamic stability. Fiberoptic bronchoscopy was performed daily, and the airway was lavaged. Lavage fluid was retained for bacterial culture.

On 1st February 2021, blood gas analysis showed that the partial pressure of oxygen (PaO_2_) was 66 mmHg. Ventilator-assisted breathing was performed for respiratory distress syndrome. Fiberoptic bronchoscopy showed yellow scab-like changes in the airway mucosa, local ulceration, no tracheal cartilage exposure, obvious congestion, and edema of the airway wall. No bacterial growth was observed in the alveolar lavage fluid at this point. On 3rd February 2021, the PaO_2_ returned to 101 mmHg. During ventilator-assisted breathing, occasional ventilator resistance was noted, and spontaneous breathing became faster. After achieving deep sedation and treating pain, the patient demonstrated good adaptability to ventilatory support. We were able to communicate with the patient to assess his condition, and we determined that he required surgical treatment for his burn wounds. Subsequently, his examination results improved, and blood transfusion and enteral and parenteral nutrition were administered.

On 4th February 2021, the 1,3-β-d-glucan/galactomannan (G/GM) test was performed using venous blood samples. The G test was positive (207.1), while the GM test was negative, and no bacteria or fungi were found in sputum culture. Bacteria were cultured using Columbia blood agar medium, chocolate medium, and McConkey agar medium at 37 °C in a carbon dioxide incubator (5–10%) in a dark environment for 3 days. Fungi were cultured using blood agar medium and Candida chromogenic medium at 37 °C for 3 days,and Sapaul medium at 25 °C for 7 days. According to the results of the G test, anti-infection drugs were adjusted to intravenous infusion of linezolid (0.6 g twice daily) + meropenem (1 g three times daily) + voriconazole (200 mg twice daily). On 7th February 2021, escharectomy, skin grafting, and Meek micrografting were performed, and the area of escharectomy and skin grafting was approximately 36% of total body surface area. After blood transfusion, anti-shock treatment, anti-sense treatment, and vasoactive drug treatment, the patient breathed quickly and produced more sputum.

**Fig. 2:**
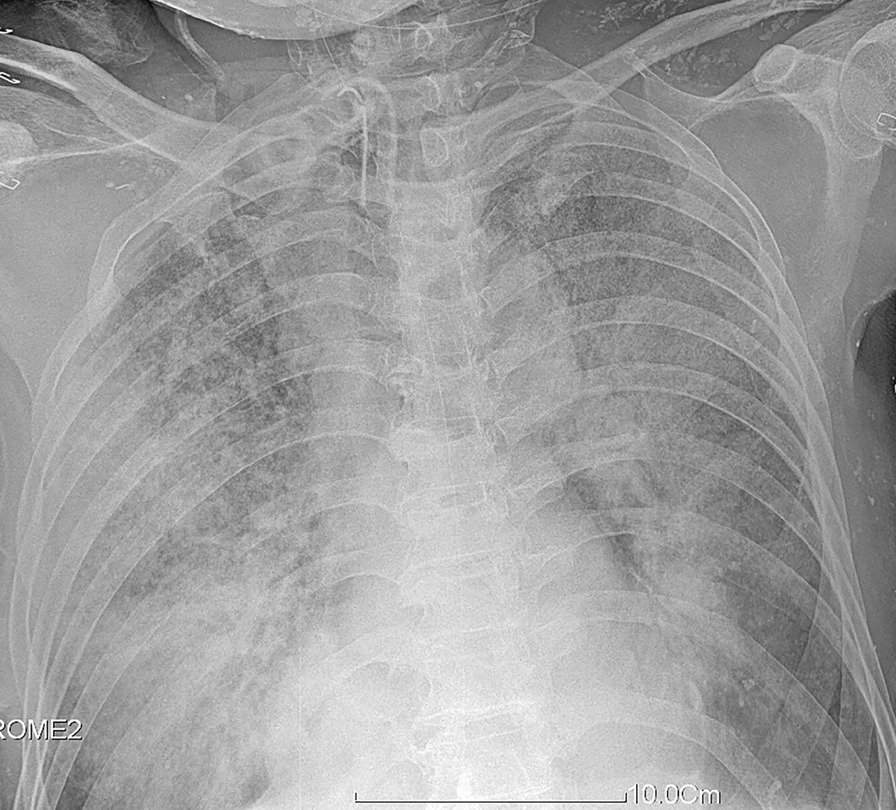
Chest X-ray showing large patellar high-density shadows in both lungs, significantly reduced pulmonary transmittance, and a widened hilum of both lungs

On 8th February 2021, chest X-ray showed a whole-lung white ground glass shadow, with large white lung imaging features (Fig. 2). Fiberoptic bronchoscopy showed severe airway mucosal damage, local tracheal cartilage exposure, no inspiratory obstruction, and expiratory blockage. Moreover, part of the necrotic airway mucosa fell off into a valve. The necrotic mucosa was stripped under fiberoptic bronchoscopy and sent for pathological examination. Alveolar lavage fluid was sent for bacterial and fungi culture.

The culture results of bacteria and fungi in sputum were negative from 4th February 2021 to 9th February 2021. On 9th February 2021, despite ventilator-assisted breathing, blood oxygen decreased. On the morning of 10th February 2021, the patient refused further treatment, requested discharge, and died on the way home.

On 10th February 2021, culture showed *Aspergillus* growth (determined from alveolar lavage fluid collected for inspection on 8th February 2021, Fig. 3). On 12th February 2021, a pathological examination showed *Aspergillus* (Fig. 4). During treatment, the patient’s temperature fluctuated around 36.5 ℃, and no high fever occurred. White blood cell count, procalcitonin, C-reactive protein, PaO_2_, partial pressure of carbon dioxide, and other data are shown in Table 1.

**Fig. 3:**
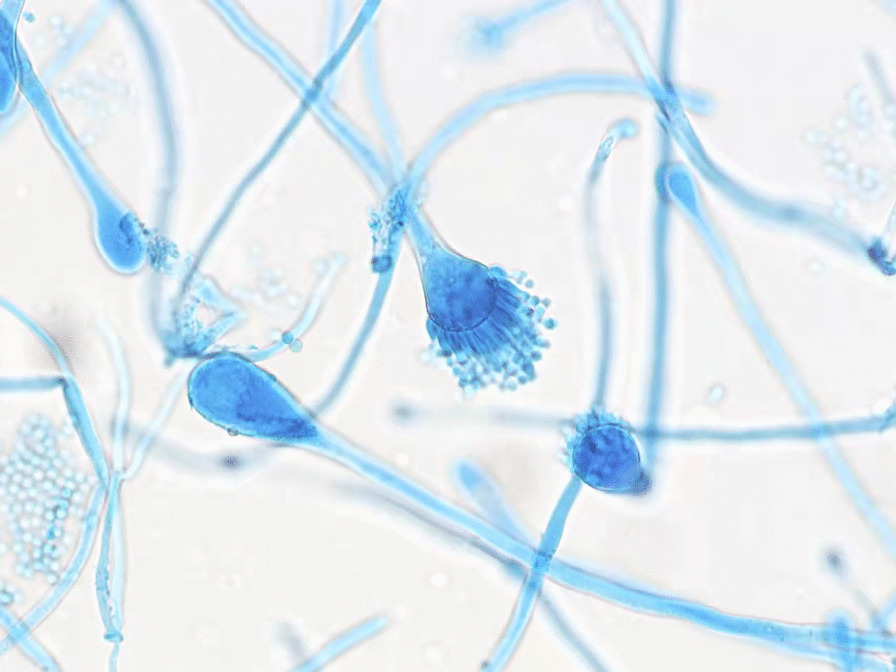
Lactic acid phenol blue staining showing *Aspergillus fumigatus*

**Fig. 4:**
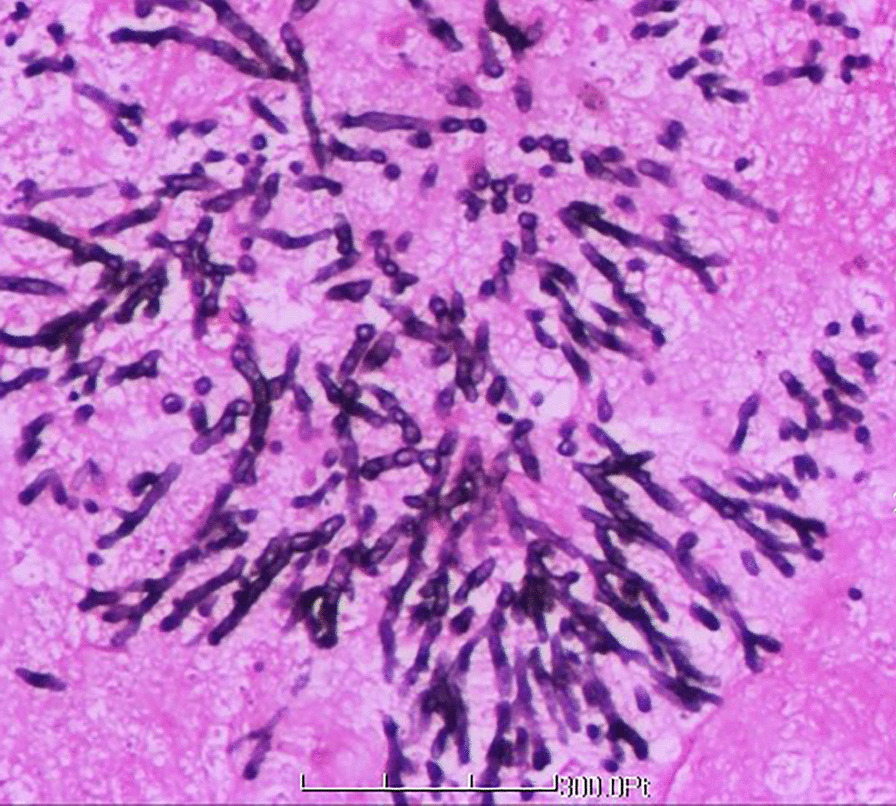
Tracheal mucosa tissues demonstrating patchy, indignant necrosis and scattered, degenerative inflammatory cells, including focal fungal hyphae. Staining: digestion periodic acid–Schiff stain (+), periodic acid–methenamine silver (+)

**Table 1:** Time, temperature, white blood cell count (WBC), procalcitonin (PCT), C-reactive protein (CRP), partial pressure of oxygen (PaO_2_), and partial pressure of carbon dioxide (PaO_2_) data table

Time	Temperature	WBC	PCT	CRP	PAO_2_	PACO_2_
2020/1/30	36.5	15.27	6.65	20.3	79.7	30
2020/1/31	37.6	12.67	12.6	34.02	73.1	39.3
2020/2/1	37.5	10.64	18.9	87.71	68.1	31.5
2020/2/2	36.8	5.71	32.53	163.66	77	37.7
2020/2/3	36.7	5.73	28.84	146.2	102	27.8
2020/2/4	36.8	4.83	15.67	117.32	104	43.2
2020/2/5	37.7	6.48	9.69	98.57	105	46
2020/2/6	36.9	10.45	4.95	122.29	83.4	35
2020/2/7	37.3	11.92	2.24	145.51	70.2	33.8
2020/2/8	36.9	8.13	5.71	86.22	75.2	33.2
2020/2/9	38.2	13.61	2.75	101.21	101	39.6
2020/2/10	36.9	16.78	7.39	276.11	61.5	91.1

When the patient was discharged from hospital, his family members said that the patient’s condition was serious, especially his lung disease. They understood that his condition progressed rapidly and that it could not be reversed. The patient’s dyspnea became progressively worse, and they felt that he may have experienced substantial pain.

## Discussion and conclusion

The key cause of severe inhalation injury in the present case was inhalation of hot furnace dust in a confined space. In addition, the patient’s age (> 60 years) and long-term smoking were high-risk factors for pulmonary aspergillosis. Combined with imaging findings and pathological results, a diagnosis was made. These results provide a basis to diagnose and treat pulmonary aspergillosis after inhalation injury. However, it cannot be ignored that inhalation injury and skin burns in this case were serious. It is undeniable that they were also the main cause of death and of the rapid decline in the patient’s condition.

Common imaging methods for pulmonary mycosis are chest X-ray and CT. Fungal laboratory examination tests include sputum culture [5], G/GM tests [6], fluorescence staining, and pathological biopsy. However, for early or atypical lesions, specimens cannot be obtained, so diagnosis is delayed [7]. At present, the most rapid diagnosis method is to advocate fluorescence staining of alveolar lavage fluid (G/GM tests) [8].

With regard to chest imaging, an increasing number of atypical cases reveal inaccuracies in imaging examinations [9]. Moreover, laboratory examinations have their own advantages and disadvantages. The positive rate of bronchoalveolar lavage fluid culture is low, but species of fungi can be distinguished clearly. Determining the G/GM ratio of bronchoalveolar lavage fluid is fast, but the species of fungi cannot be directly identified. Fluorescence staining of bronchoalveolar lavage fluid can also be performed quickly, and bacterial species can be identified, but the positive rate still needs to be improved. In recent years, lavage fluid/sputum next-generation sequencing has become an important means to identify the source of infection [10], and it can rapidly diagnose fungal infection. However, it is expensive, and samples often need to be sent for professional inspection. The strains of fungal infection are *Candida*, *Aspergillus*, and *Mucor*, with pulmonary *Aspergillus* infection demonstrating an upward trend [11, 12]. The 2017 ESCMID-ECMM-ERS guideline [13] emphasized the importance of imaging and bronchoalveolar lavage fluid examination. In the present case, the patient completed imaging studies from the onset of inhalation injury, followed by daily microbiological culture of airway lavage fluid with fiberoptic bronchoscopy and intermittent G/GM examinations, which did not explicitly reveal pulmonary aspergillosis. It was not until severe hypoxemia that chest radiography showing a big white lung, and removal of the mucous membrane by fiberoptic bronchoscopy confirmed the diagnosis of pulmonary *Aspergillus* infection. It is suggested that in addition to chest imaging and comprehensive and detailed laboratory examination of bronchoalveolar lavage fluid, airway mucosa biopsy is also very important to identify *Aspergillus* infection in patients with severe inhalation injury.

When selecting antifungal drugs, commonly used antifungal drugs include azoles and echinocandins, amongst others [14]. In the past, oral preparations of fluconazole were mostly used for *Candida* infection. However, as the infection rates of *Aspergillus* and *Mucor* increase, more effective broad-spectrum antifungal drugs are needed, and itraconazole (as well as voriconazole, micafungin, and caspofungin) should be considered [15, 16]. Itraconazole and voriconazole are increasingly recommended [13] and used [12], and there have been successful cases of atomization with amphotericin [17, 18]. Many scholars have pointed out that inhalation of amphotericin can relieve airway spasm [19, 20], reduce the frequency of asthma [21], control the spread of *Aspergillus* infection [22], and provide superior security [23]. However, some people have proposed that there is no effective antifungal agent for trauma at present [24], which is also the main reason for the increased incidence of fungal infections each year. At present, new drugs targeting host factors are also under development [25], which aim to substantially solve pulmonary aspergillosis. In view of the problem of ineffective anti-infection treatment in the present case, a large area of burns and severe inhalation injury cannot be ignored. However, we think that intravenous voriconazole combined with amphotericin atomization may benefit such patients.

It is of vital importance to establish an atlas of airway fungal infection as soon as possible. Such an atlas could describe the location, range, secretion, bleeding, and airway stenosis, amongst other factors. This atlas should describe the manifestations of different bacterial species, and it should enable the direction of diagnosis to be clarified as soon as possible, allowing anti-infection treatment to be administered. This atlas would resemble the atlas of skin diseases, which provides accurate clinical case pictures. Although not like the gold standard of histopathology, this method would have the advantage of benefiting patients.

We summarized the following microscopic characteristics of aspergillosis. These characteristics were combined with our previous experience in the diagnosis and treatment of pulmonary aspergillosis, as well as with a literature summary [26, 27]. First, under fiberoptic bronchoscopy, airway mucosal congestion and swelling, stenosis, and occlusion are obvious. Second, leukoplakia can be seen locally in the airway wall mucosa, and pseudomembrane formation is observed in severe cases, which is accompanied by ulceration. Moreover, lesions bleed easily when touched. Third, there is no obvious airway secretion without bacterial infection. After severe inhalation injury, the airway wall is seriously damaged and necrotic. The shedding of necrotic tissue leads to airway obstruction or valve formation, which makes the condition worsen rapidly. Therefore, a simple, recyclable, antibacterial, and supportive tracheal stent is urgently needed for “dressing changes” in the burned airway. Absorbable tracheal stents may help to prevent foreign bodies from blocking the airway.

In conclusion, for patients with severe inhalation injury, it is necessary to recognize the possibility of early *Aspergillus* infection. Moreover, early diagnosis and targeted anti-*Aspergillus* therapy are important to save patients’ lives.

## Data Availability

The datasets used and/or analyzed during the current study are available from the corresponding author on reasonable request. The data and materials, including all the clinical data of the patients are included within the article

## Abbreviations

CT: Computed tomography
TBSA: Total body surface area
G test: 1,3-β-D-glucan test
GM test: Galactomannan test
ESCMID: European Society of Clinical Microbiology and Infectious Diseases
ECMM: European Confederation of Medical Mycology
ERS: European Respiratory Society
